# Prevalence, practices, and maternal perceptions of traditional uvulectomy among sudanese children and adolescents: A community-based cross-sectional study

**DOI:** 10.1371/journal.pone.0328341

**Published:** 2026-07-16

**Authors:** Jaber Hamad Jaber Amin, Mohamedelmustafa Yahya Mohamed Eldouma, Sakshi Kumari, Ahmed Alshafei Elmahi Ahmed, Alshafee Mohammed Jebreel Sulyman, Amna A. Eltayeb, Asjad Zainalabdeen Ahmed, Huyam Elsir Fadlelseed Babiker, Tanzeel Mohamedain Abuelgasim Abdalla, Dania A. Elsiddig, Majdy Jailany Alamin Abdelgadir, Nooralsham Abdalla Adam Yousif, Israa Osman Ali Alhassan, Zainab Salih Ahmed Adam, Ahmed Yahia Mahadi Babiker, Mohammed Hammad Jaber Amin, Leina Elomeiri

**Affiliations:** 1 University of Sinnar, Sinnar, Sudan; 2 University of Khartoum, Khartoum, Sudan; 3 Shimoga Institute of Medical Sciences, Shimoga, India; 4 National Ribat University, Khartoum, Sudan; 5 Abdulatif Alhamad University of Technology, Merowe, Sudan; 6 University of khartoum, khartoum, Sudan; 7 University of Gadarif, Gadarif, sudan; 8 University of Gezira, Gezira, Sudan; 9 National Ribat University, Khartoum, Sudan; 10 University of Khartoum, Khartoum, Sudan; 11 Abdulatif Alhamad University of Technology, Merowe, Sudan; 12 Omdurman Ahlia University, Khartoum, Sudan; 13 Abdulatif Alhamad University of Technology, Merowe, Sudan; 14 University of Al-Neelain, Khartoum, Sudan; 15 University of Khartoum, Khartoum, Sudan; 16 Alzaiem Alazhari University, Khartoum, Sudan; 17 University of Khartoum, Khartoum, Sudan; Menzies School of Health Research: Charles Darwin University, AUSTRALIA

## Abstract

**Background:**

Traditional uvulectomy (TU) is the removal of the uvula, either partially or totally, by the traditional healer. It is prevalent in sub-Saharan Africa, driven by cultural beliefs and perceived therapeutic benefits, despite the risks of complications such as bleeding, infections, and death, and limited data exist. Our study aimed to assess the prevalence and practice of traditional uvulectomy among Sudanese children and adolescents, along with maternal perceptions, and identify associated risk factors.

**Method:**

A descriptive community-based cross-sectional study was conducted between January and April 2025 across more than ten states on mothers of children and adolescents (≤18 years). Data were collected via face-to-face interviews using a validated structured questionnaire to assess the socio-demographics, TU perception regarding TU, practice pattern, and reported complications. A convenience sampling technique was used. Statistical analyses were performed using R software, with p < 0.05 considered statistically significant.

**Results:**

A total of 1,135 mothers of children and adolescents (≤18 years) were interviewed face-to-face. The overall prevalence of TU was 15%, with higher rates among children <6 years (16%) and adolescents aged 12–18 years (17%). Maternal belief in TU varied significantly by child age (p < 0.001). Commonly cited reasons included breastfeeding difficulties (18%), cultural tradition (16%), and failure to thrive (13%). Mothers (45%) and grandmothers (41%) were the primary decision-makers, while traditional healers performed 55% of procedures, frequently using unsterilized instruments. Reported complications included fever (35%), feeding difficulties (21%), and bleeding (13%). TU practice was significantly associated with younger maternal age, lower educational attainment, higher parity, and strong adherence to tribal traditions (all p < 0.001).

**Conclusion:**

Traditional uvulectomy remains prevalent in Sudan due to deeply rooted cultural beliefs, intergenerational influence, and gaps in healthcare access. Targeted, culturally sensitive interventions engaging tribal leaders, grandmothers, and traditional healers, alongside improved health education and service accessibility, are essential to reduce associated child health risks.

## Introduction

Traditional uvulectomy (TU) refers to the partial or complete removal of the uvula, typically performed by traditional healers. The uvula is a small, fleshy structure suspended from the posterior margin of the soft palate between the palatine tonsils. It plays an important role in speech articulation, lubrication of the oropharyngeal mucosa, immune defense through antibody-rich secretions, and coordination with the soft palate to prevent nasopharyngeal regurgitation and aspiration during swallowing [1–3]. Developmentally, the uvula forms through fusion of the soft palate during the 11th week of gestation and is supplied by branches of the ascending pharyngeal and palatine arteries, with innervation from the glossopharyngeal and lesser palatine nerves [4–6].

In many low- and middle-income settings, traditional healers are trusted providers of healthcare, offering culturally accepted remedies and procedures that are transmitted across generations [7,8]. Although uvulectomy is rarely performed within formal healthcare settings, except as part of specific surgical interventions such as uvulopalatoplasty or adenotonsillectomy, it remains widely practiced as a traditional procedure [9,10]. TU is prevalent across sub-Saharan Africa, including Nigeria, Kenya, Sierra Leone, Tanzania, Ethiopia, South Africa, and Sudan [11–17], and has also been reported in Israel, Saudi Arabia, and other Middle Eastern countries [11,18,19]. Cultural beliefs, family traditions, and socioeconomic constraints strongly perpetuate the practice; for instance, previous studies report that up to one-third of mothers endorse TU, with family tradition cited as a major motivating factor [17]. Low educational attainment, limited access to healthcare services, and broader structural inequities further reinforce reliance on traditional practices [20,21].

The perceived rationale for TU varies widely. Parents often attribute childhood illnesses to the uvula and fear complications such as airway obstruction or sudden death. Commonly cited indications include feeding and swallowing difficulties, recurrent throat infections, failure to thrive, recurrent fever, chronic cough, and feeding aversion [3,20]. In some settings, male children, frequently viewed as carriers of family lineage, are more likely to receive immediate traditional interventions, including uvulectomy, when ill [3]. The procedure is typically performed without anesthesia using unsterilized instruments, and herbal substances are often applied to the wound [22]. As a result, TU is associated with severe and potentially life-threatening complications, including hemorrhage, anemia, bacterial and viral infections (including tetanus and HIV), abscess formation, aspiration, upper airway obstruction, and death [9,23,24]. Additional reported complications include jaundice, prolonged pain, voice changes, sleep disturbances, regurgitation of breast milk, and cavernous sinus thrombosis [17]. These adverse outcomes frequently necessitate increased healthcare utilization, such as antibiotic therapy, intravenous fluids, oxygen supplementation, blood transfusion, and phototherapy, placing additional strain on already limited health systems [3,21,23]. In Ethiopia, for example, approximately 20% of children undergo TU, with 15% experiencing severe complications requiring hospitalization [22].

Qualitative and quantitative studies have consistently identified cultural beliefs, strong family and community pressure, ease of access to traditional healers, limited awareness of medical risks, and geographical barriers to healthcare facilities as key drivers of TU [21]. In Sudan, a hospital-based study reported that 17.9% of children under five years had undergone uvulectomy, with maternal age, educational level, and regional background independently associated with the practice [13]. However, nationally representative community-based data, particularly including adolescents and maternal perceptions, remain scarce.

Therefore, this study aims to assess the prevalence and practice of traditional uvulectomy among Sudanese children and adolescents, explore maternal perceptions surrounding the practice, and identify associated sociodemographic and cultural risk factors. The findings are intended to inform culturally sensitive public health interventions, policy development, and health education strategies aimed at reducing preventable harm and promoting appropriate health-seeking behavior.

## Methodology

### Study design and setting

This descriptive, community-based cross-sectional study assessed the prevalence and practice of traditional uvulectomy (TU) among Sudanese children and adolescents, as well as maternal perceptions regarding the practice. The study was conducted between 1 January and 20 April 2025 across more than ten Sudanese states.

### Study population and sampling

The study included mothers who had at least one child or adolescent aged ≤18 years and who consented to participate. Mothers who declined participation or whose children were outside the specified age range were excluded. A convenience sampling technique was employed due to logistical constraints and population displacement.

### Data collection

Data were collected through face-to-face interviews conducted in private settings by trained medical students using a structured questionnaire adapted from previously validated studies [15,25,31]. The questionnaire was initially developed in English, translated into the Arabic language to ensure comprehension, and back-translated to confirm accuracy. Interviewers received standardized training on questionnaire administration, ethical conduct, and non-leading interviewing techniques.

The questionnaire captured information on sociodemographic characteristics (maternal age, education level, residence, and tribal affiliation); maternal knowledge and perceptions regarding traditional uvulectomy; practice-related characteristics, including the practitioner, instruments used, and timing of the procedure; and reported complications following uvulectomy.

To minimize information bias, interviewers provided standardized explanations of each item without prompting responses, and the data collection process was closely supervised by a senior researcher.

### Statistical analysis

Statistical analysis was performed using R software (version 4.4.2), utilizing the tidyverse and related packages. Categorical variables were summarized as frequencies and percentages and compared using the Pearson chi-squared test or Fisher’s exact test, as appropriate. Continuous variables were summarized using medians and interquartile ranges (IQRs) and compared using the Wilcoxon rank-sum test. A p-value < 0.05 was considered statistically significant.

### Ethical considerations

Ethical approval was obtained from the General Administration of Health Systems and Research, Federal Ministry of Health, River Nile State, Sudan. The study was conducted in accordance with the Declaration of Helsinki. Written informed consent was obtained from all participants prior to data collection. Participant confidentiality was ensured through data anonymization, and participants were informed of their right to withdraw from the study at any time without consequence.

## Results

### Sociodemographic factors

The study included 1,135 mothers from Sudan. The median age of the participants was 28 years (IQR: 17, 51). The mothers were well-educated, with a significant proportion holding graduate (38%) or postgraduate (7.6%) degrees. Most respondents resided in Khartoum (22%) or Northern states (21%). The majority of households (61%) were classified as having a moderate income (Table 1).

**Table 1:** Sociodemographic Characteristics of Mothers and Children Included in the Study (N = 1,135).

Characteristic	N = 1,135^1^
**Mother’s Age**	28 (17, 51)
**Mother education**	
Illiterate	105 (9.3%)
Informal Education	86 (7.6%)
Primary	193 (17%)
Secondary	236 (21%)
Graduate	429 (38%)
Postgraduate	86 (7.6%)
**Residence State**	
Khartoum	252 (22%)
Northern	242 (21%)
Kassala	199 (18%)
River Nile	134 (12%)
Al Jazeera	54 (4.8%)
Al Qadarif	37 (3.3%)
Red Sea	22 (1.9%)
Other States	36 (3.2%)
Outside Sudan	159 (14%)
**Age of your oldest child** (Median & IQR)	12 (6, 21)
**How old were you when you gave birth to your oldest child?**	22.0 (19.0, 25.0)
**How many children do you have?**	3.00 (2.00, 5.00)
**Age of your youngest child**	
< 1 year	50 (4.4%)
1-3 years	398 (35%)
3-6 years	279 (25%)
6-12 years	222 (20%)
12-18 years	186 (16%)
**Sex of the youngest child**	
Male	600 (53%)
Female	535 (47%)
**Monthly income of the family**	
Low income	360 (32%)
Moderate income	687 (61%)
High income	81 (7.2%)
**The mother work**	342 (30%)
**Monthly income of the mother**	
Low income	181 (39%)
Moderate income	266 (57%)
High income	18 (3.9%)
^1^Median (Q1, Q3); n (%)	

### Awareness, prevalence, and beliefs regarding UVulectomy

Overall, 57% of mothers had heard of the practice, and 35% reported that it was present within their tribe. Younger mothers (under 35) were significantly more likely to be aware of the practice (68% vs. 42%, p < 0.001) and to believe in it (34% vs. 11%, p < 0.001) compared to older mothers. Additionally, mothers whose youngest child was in the 12–18 years age group showed the highest level of belief (37%), a significantly greater proportion than those with children aged 6–12 years (16%) (p < 0.001) (Table 2).

**Table 2:** Prevalence and perception of the traditional uvulectomy practice in children among the mothers, categorized by their age groups and their youngest child’s age and sex.

	Age of the Mothers (years)					Youngest Child Age (years)							Youngest Child Sex					
Characteristic	< 35 N = 669^1^	95% CI	≥ 35 N = 441^1^	95% CI	p-value^2^	< 6 N = 727^1^	95% CI	6-12 N = 222^1^	95% CI	12-18 N = 186^1^	95% CI	p-value^2^	M N = 600^1^	95% CI	F N = 535^1^	95% CI	p-value^2^	Sum N = 1,135^1^
**Heard of the traditonal uvulectomy practice**	456 (68%)	64%, 72%	185 (42%)	37%, 47%	**<0.001**	407 (56%)	52%, 60%	126 (57%)	50%, 63%	119 (64%)	57%, 71%	0.14	362 (60%)	56%, 64%	290 (54%)	50%, 58%	**0.037**	652 (57%)
**Your child had his/her uvula removed**	130 (20%)	17%, 23%	35 (8.1%)	5.8%, 11%	**<0.001**	115 (16%)	13%, 19%	19 (8.7%)	5.5%, 13%	31 (17%)	12%, 23%	**0.020**	111 (19%)	16%, 22%	54 (10%)	7.8%, 13%	**<0.001**	165 (15%)
**Will schedule your child for uvulectomy**	126 (19%)	16%, 23%	31 (7.4%)	5.1%, 10%	**<0.001**	120 (17%)	14%, 20%	13 (6.2%)	3.5%, 11%	25 (14%)	9.4%, 20%	**<0.001**	104 (18%)	15%, 21%	54 (10%)	8.0%, 14%	**<0.001**	158 (14%)
**Uvulectomy is practiced in your tribe**	292 (47%)	43%, 52%	69 (17%)	14%, 21%	**<0.001**	230 (33%)	30%, 37%	74 (38%)	31%, 45%	61 (40%)	32%, 48%	0.2	224 (41%)	36%, 45%	141 (29%)	25%, 33%	**<0.001**	365 (35%)
**Totally believe in these practices**	169 (34%)	30%, 38%	35 (11%)	7.8%, 15%	**<0.001**	139 (24%)	21%, 28%	25 (16%)	11%, 23%	42 (37%)	28%, 46%	**<0.001**	124 (28%)	24%, 32%	82 (21%)	17%, 25%	**0.025**	206 (24%)

1 n (%)

2 Pearson’s Chi-squared test

Abbreviation: CI = Confidence Interval

The overall prevalence of the practice was 15%, with 165 children having undergone the procedure. The practice was significantly more common among male children (19%) than female children (10%) (p < 0.001). Prevalence also varied by the child’s age group, with higher rates observed in children under 6 years (16%) and those aged 12–18 years (17%), compared to a lower rate in the 6–12 years age group (8.7%) (p = 0.020) (Table 2).

### Reasons for uvulectomy and perceptions of harm

Mothers who had a child undergo uvulectomy reported specific clinical symptoms far more frequently than mothers without direct experience. The primary reasons given by the practicing group were difficulty breastfeeding (41%), vomiting (38%), and loss of appetite (37%). These reasons were all significantly more common in the group that practiced uvulectomy (p < 0.001 for all). Furthermore, among mothers whose child had a uvulectomy, a majority believed the uvula causes oropharyngeal blockage (76%), that uvulectomy is a good treatment (84%), and that the uvula’s presence is harmful if not removed (70%) (Table 3).

**Table 3:** Maternal Beliefs and Perceptions Regarding Traditional Uvulectomy categorized by if they had a child with his/her uvula removed or not.

Characteristic	Overall N = 1,123^1^	95% CI	No child with uvula removed N = 958^1^	95% CI	Had a child with uvula removed N = 165^1^	95% CI	p-value^2^
**Reasons for uvulectomy practice**							
Cultural reasons/family tradition	175 (16%)	14%, 18%	139 (15%)	12%, 17%	36 (22%)	16%, 29%	**0.017**
Loss of appetite	136 (12%)	10%, 14%	75 (7.8%)	6.2%, 9.8%	61 (37%)	30%, 45%	**<0.001**
Difficulty breastfeeding	203 (18%)	16%, 20%	135 (14%)	12%, 16%	68 (41%)	34%, 49%	**<0.001**
Failure to gain weight	146 (13%)	11%, 15%	104 (11%)	9.0%, 13%	42 (25%)	19%, 33%	**<0.001**
Vomiting	143 (13%)	11%, 15%	81 (8.5%)	6.8%, 10%	62 (38%)	30%, 45%	**<0.001**
Preventive of illness or choking	59 (5.3%)	4.1%, 6.8%	42 (4.4%)	3.2%, 5.9%	17 (10%)	6.3%, 16%	**0.002**
Delay in teething	1 (<0.1%)	0.00%, 0.58%	1 (0.1%)	0.01%, 0.68%	0 (0%)	0.00%, 2.8%	>0.9
Cough	82 (7.3%)	5.9%, 9.0%	52 (5.4%)	4.1%, 7.1%	30 (18%)	13%, 25%	**<0.001**
Growth retardation	1 (<0.1%)	0.00%, 0.58%	0 (0%)	0.00%, 0.50%	1 (0.6%)	0.03%, 3.8%	0.15
Fever	22 (2.0%)	1.3%, 3.0%	14 (1.5%)	0.83%, 2.5%	8 (4.8%)	2.3%, 9.7%	**0.009**
Diarrhea	24 (2.1%)	1.4%, 3.2%	9 (0.9%)	0.46%, 1.8%	15 (9.1%)	5.4%, 15%	**<0.001**
Other	30 (2.7%)	1.8%, 3.8%	27 (2.8%)	1.9%, 4.1%	3 (1.8%)	0.47%, 5.6%	0.6
**Does the uvula cause oropharyngeal blockage in children?**							**<0.001**
No	295 (37%)	34%, 40%	276 (43%)	39%, 47%	19 (12%)	7.4%, 18%	
Not sure	297 (37%)	34%, 41%	277 (43%)	39%, 47%	20 (12%)	7.9%, 19%	
Yes	208 (26%)	23%, 29%	86 (13%)	11%, 16%	122 (76%)	68%, 82%	
**Is uvulectomy a good treatment for the above?**							**<0.001**
No	277 (34%)	30%, 37%	267 (40%)	36%, 44%	10 (6.2%)	3.2%, 11%	
Not sure	301 (36%)	33%, 40%	285 (43%)	39%, 47%	16 (9.9%)	5.9%, 16%	
Yes	248 (30%)	27%, 33%	112 (17%)	14%, 20%	136 (84%)	77%, 89%	
**If the uvula is not removed, will its presence harm the child?**							**<0.001**
No	393 (47%)	44%, 51%	371 (55%)	52%, 59%	22 (14%)	8.9%, 20%	
Not sure	237 (28%)	25%, 32%	210 (31%)	28%, 35%	27 (17%)	11%, 24%	
Yes	202 (24%)	21%, 27%	89 (13%)	11%, 16%	113 (70%)	62%, 77%	

^1^n (%)

^2^Pearson’s Chi-squared test; Fisher’s exact test

Abbreviation: CI = Confidence Interval

### Decision-making, procedure details, and complications

**Fig 1:**
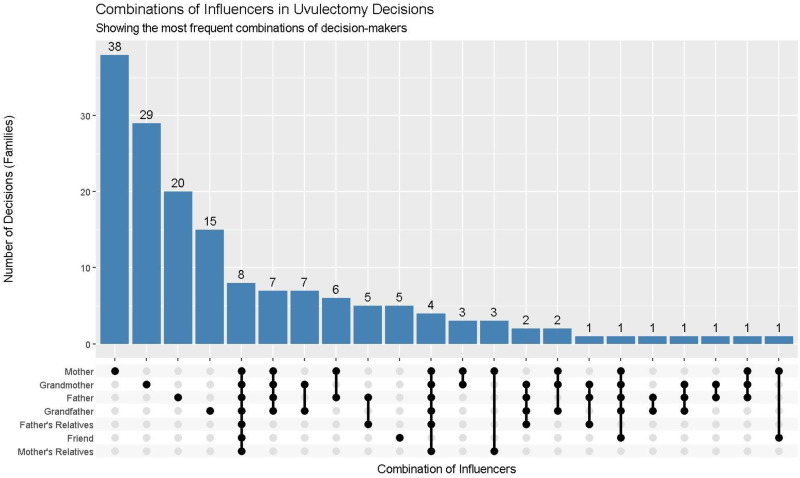
Combinations of influencers in uvulectomy decisions among the participants (n = 135).

The decision to perform a uvulectomy was predominantly influenced by the mothers themselves (45%) and grandmothers (41%). Fathers also played a significant role in 35% of cases (Table 4, Fig 1). The procedure was most commonly performed by a traditional healer (55%), followed by the mother or other relatives (19%) and older women in the community (18%) (Table 4).

**Table 4:** Decision-Makers and Providers Involved in Traditional Uvulectomy Practices.

Characteristic	N = 165^1^
**Who decided to perform the procedure?**	
Mother	74 (45%)
Grandmother	67 (41%)
Father	58 (35%)
Grandfather	48 (29%)
Father’s relatives	20 (12%)
Mother’s relatives	15 (9.1%)
Friend	15 (9.1%)
Other	5 (3.0%)
**Who performed the procedure?**	
Traditional healer (i.e., Fakky or Baseer)	90 (55%)
Mother or relatives	32 (19%)
Older women in the community	29 (18%)
Medical assistant	10 (6.1%)
Other	9 (5.5%)
^1^n (%)	

The methods used were often crude, with non-sterile instruments like flat irons (25%) and hooked nails (19%) being frequently reported. Traditional remedies were common for post-procedure care, with acacia (44%) being the most used substance (Table 5).

**Table 5:** Methods, medications, and reported complications of performed uvulectomy among the study participants.

Characteristic	N = 165^1^
**Methods used**	
Flat iron	41 (25%)
Nail Hooked	32 (19%)
Bar Razor Blade	25 (15%)
Dental Instrument	23 (14%)
Don’t know what method was used	46 (28%)
String	1 (50%)
Hand	1 (50%)
**Traditional remedies used after uvulectomy**	
Acacia (i.e., Qarad)	72 (44%)
Rose geraniums (i.e., a’atroon)	34 (21%)
Other remedies	21 (13%)
None	45 (27%)
**How soon after uvulectomy did you breastfeed/feed your child?**	
Immediately	78 (49%)
Later that day	53 (33%)
One day post-operation	16 (10%)
Two days post-operation	4 (2.5%)
More than three days post-operation	9 (5.6%)
**Complications reported**	
Fever	58 (35%)
Difficulty in feeding	35 (21%)
Bleeding	22 (13%)
Secondary infection	8 (4.8%)
Tetanus	2 (1.2%)
Other	7 (4.2%)
^1^n (%)	

### Factors associated with the practice of uvulectomy

Mothers who practiced uvulectomy were significantly younger (median age 22 vs. 28, p < 0.001) and had a lower level of formal education. Notably, 52% of mothers in the practicing group were either illiterate or had only informal education, compared to just 11.1% in the non-practicing group (p < 0.001). Furthermore, the practice was strongly associated with having more children and the child being male (p < 0.001 for both) (Table 6). Furthermore, a multiple logistic regression model confirmed these findings, identifying several independent predictors (Table 7). The strongest predictor was a mother’s intention to schedule the procedure, which increased the odds of the practice by 60-fold (OR = 60.0; 95% CI: 29.5, 131; p < 0.001). The presence of the practice within the mother’s tribe was also a powerful predictor, increasing the odds by over 6 times (OR = 6.38; 95% CI: 2.89, 14.8; p < 0.001). Having more children was also a significant risk factor (OR = 1.24 per child; p = 0.005). Conversely, higher levels of education were strongly protective, with a secondary school education (OR = 0.33) and a graduate degree (OR = 0.24) significantly reducing the odds of the practice compared to being illiterate.

**Table 6:** The association between demographic characteristics and traditional uvulectomy.

Characteristic	No N = 958^1^	95% CI	Yes N = 165^1^	95% CI	p-value^2^
**Mother’s Age**	28 (17, 51)	34, 37	22 (22, 33)	26, 31	**<0.001**
**Mother education**					**<0.001**
Illiterate	71 (7.4%)	5.9%, 9.3%	34 (21%)	15%, 28%	
Informal	35 (3.7%)	2.6%, 5.1%	51 (31%)	24%, 39%	
Primary	151 (16%)	14%, 18%	41 (25%)	19%, 32%	
Secondary	206 (22%)	19%, 24%	25 (15%)	10%, 22%	
Graduate	411 (43%)	40%, 46%	13 (7.9%)	4.4%, 13%	
Postgraduate	84 (8.8%)	7.1%, 11%	1 (0.6%)	0.03%, 3.8%	
**Age of her oldest child**	11 (6, 20)	13, 14	16 (10, 25)	16, 19	**<0.001**
**Mother’s age when she had her first child**	22.0 (19.0, 26.0)	22, 23	20.0 (17.0, 22.0)	20, 21	**<0.001**
**Total number of children**	3.00 (2.00, 5.00)	3.2, 3.5	4.00 (3.00, 6.00)	4.3, 5.0	**<0.001**
**Age of her youngest child**					**0.048**
< 1 year	40 (4.2%)	3.0%, 5.7%	10 (6.1%)	3.1%, 11%	
1-3 years	333 (35%)	32%, 38%	64 (39%)	31%, 47%	
3-6 years	234 (24%)	22%, 27%	41 (25%)	19%, 32%	
6-12 years	199 (21%)	18%, 24%	19 (12%)	7.3%, 18%	
12-18 years	152 (16%)	14%, 18%	31 (19%)	13%, 26%	
**Gender of the youngest child**					**<0.001**
Male	482 (50%)	47%, 54%	111 (67%)	59%, 74%	
Female	476 (50%)	46%, 53%	54 (33%)	26%, 41%	
**Monthly income of the family**					0.4
Low income	298 (31%)	28%, 34%	60 (36%)	29%, 44%	
Moderate income	585 (62%)	58%, 65%	95 (58%)	50%, 65%	
High income	68 (7.2%)	5.6%, 9.0%	10 (6.1%)	3.1%, 11%	
**Mother works** (yes).	271 (28%)	25%, 31%	68 (41%)	34%, 49%	**<0.001**
**Monthly income of the mother**					>0.9
Low income	145 (39%)	34%, 44%	34 (38%)	28%, 49%	
Moderate income	212 (57%)	52%, 62%	52 (58%)	47%, 68%	
High income	14 (3.8%)	2.2%, 6.4%	4 (4.4%)	1.4%, 12%	
**Will you schedule your child for uvulectomy?**	33 (3.5%)	2.4%, 4.9%	124 (84%)	77%, 90%	**<0.001**
**Presence of uvulectomy practice in your tribe**	213 (25%)	22%, 28%	151 (92%)	86%, 95%	**<0.001**

^1^Median (Q1, Q3); n (%)

^2^Wilcoxon rank sum test; NA; Pearson’s Chi-squared test; Fisher’s exact test

Abbreviation: CI = Confidence Interval

**Table 7:** Multiple logistic regression predicting traditional uvulectomy practice based on socio-demographics and perception of the mothers.

Characteristic	OR	95% CI	p-value
**Mothers’ age groups**			
< 35 years	—	—	
≥ 35 years	1.56	0.73, 3.35	0.3
**Mother education**			
Illiterate	—	—	
Informal	2.81	0.92, 8.73	0.070
Primary	0.64	0.23, 1.74	0.4
Secondary	0.33	0.12, 0.93	**0.038**
Graduate	0.24	0.07, 0.80	**0.021**
Postgraduate	0.27	0.01, 1.80	0.2
**How many children do you have?**	1.24	1.06, 1.45	**0.005**
**Youngest child**			
< 6 years	—	—	
6-12 years	0.50	0.18, 1.31	0.2
12-18 years	1.56	0.65, 3.77	0.3
**Gender of the youngest child**			
Male	—	—	
Female	0.58	0.30, 1.12	0.11
**Monthly income of the family**			
Low income	—	—	
Moderate income	2.01	1.00, 4.10	0.052
High income	1.01	0.24, 4.13	>0.9
**Will she schedule her child for uvulectomy?**			
No	—	—	
Yes	60.0	29.5, 131	**<0.001**
**Presence of uvulectomy practice in her tribe**			
No	—	—	
Yes	6.38	2.89, 14.8	**<0.001**

Abbreviations: CI = Confidence Interval, OR = Odds Ratio

## Discussion

Traditional uvulectomy remains a significant public health concern in Sudan. In the present study, 15% of children and adolescents had undergone uvulectomy, confirming that the practice persists despite increasing access to modern healthcare. The observed prevalence varied significantly according to child sex, age group, and maternal age, highlighting the strong influence of sociocultural and demographic factors.

Sudan’s prevalence rate falls between that reported in Tanzania (3.6%) [17] and higher rates observed in Southwest Ethiopia (61.9%) [7], Arbaminch, Ethiopia (36.6%) [26], Ekiti, Nigeria (26.9%) [27], and Tanzania (3.6%) [28]. These regional differences likely reflect variations in cultural beliefs, access to healthcare services, educational attainment, and socioeconomic conditions.

A bimodal age distribution was observed, with peaks among children under six years (16%) and adolescents aged 12–18 years (17%). The age distribution matches the age pattern seen in Southwest Ethiopia [7]. The age distribution suggests health reasons and social reasons—relief of symptoms in infants and keeping tradition in adolescents. In our work, we saw that the practice does things at different ages. The practice serves social and cultural roles at different stages of growth.

Cultural beliefs and perceived therapeutic benefits remain the principal drivers of the practice. In this study, mothers cited breastfeeding difficulties (18%), cultural traditions (16%), and failure to thrive (13%) as primary reasons for uvulectomy, consistent with findings from Ethiopia and Nigeria [7,17,23,27]. Nearly 60% of cases were driven by belief-based reasoning. Thirty percent of mothers believed the procedure was beneficial, while 36% reported uncertainty, and 24% believed the uvula itself could cause harm. Comparable misconceptions have been documented elsewhere; for example, 90% of participants in Tanzania believed illness originated from the uvula, and 64.7% of respondents in Nigeria held similar beliefs. [27,28]. Frequently cited reasons for traditional uvulectomy in Ethiopia were to prevent swelling, pus, and rupture of the uvula, for better care, prevention of sore throats and coughs, religion, and culture. [29] Multivariable analysis demonstrated that maternal intention was the strongest predictor of uvulectomy (OR ≈ 60), followed by tribal affiliation (OR ≈ 6.4). These findings indicate that deeply embedded social norms outweigh individual educational attainment in shaping behavior. Even when mothers possessed formal education, it was insufficient to counteract strong communal beliefs.

Even though many mothers hold strong beliefs regarding traditional uvulectomy, the findings reveal substantial knowledge gaps concerning its risks. In this study, 34% of mothers reported that traditional uvulectomy does not work, 26% believed that the uvula could obstruct the airway, and 37% were uncertain about its effects. This level of uncertainty, particularly the one-third of mothers who were unsure, highlights a critical opportunity for targeted public health education. The proportion of mothers expressing fear of harm related to the uvula (24%) was lower than that reported by Adamu et al., where 68.3% of respondents believed that traditional uvulectomy was beneficial for managing vomiting and swallowing difficulties [27,30]. These findings underscore the need for focused educational interventions addressing misconceptions about the function of the uvula and the risks associated with its removal.

Family decision-making dynamics further shape the persistence of this practice. Mothers (45%) and grandmothers (41%) were the primary decision-makers, while traditional healers performed 55% of procedures, a considerably lower proportion than the 76.5% reported in Nigeria [18]. This pattern reflects the transmission of beliefs within families rather than sole reliance on traditional practitioners. Similar dynamics have been reported in Eritrea and Ethiopia, where elder family members play a central role in healthcare decisions [17,30]. The strong influence of grandmothers, in particular, underscores the importance of including elder women in culturally sensitive behavioral change interventions.

The decision makers in our study ranged in age between 18 and 52 years, with a median age of 33 years. Educational attainment varied widely: 35% had university-level education, 21% completed secondary school, 18% completed primary education, and 10% were unable to read or write. Despite this relatively broad educational profile, traditional practices persisted even among educated individuals, indicating that cultural norms often outweigh formal knowledge. Similar patterns were observed in Gondar Town, Ethiopia, where 11.6% of mothers were illiterate, 38.7% had completed secondary education, and only 3.6% had attained college education [7]. In Sokoto State, Nigeria, 43.8% of participants had received some level of education, while only 2.8% lacked formal schooling [27]. Furthermore, Adamu A et al. reported that most respondents were unemployed and that very few had never attended school [30]. In the present study, 70% of respondents were unemployed, reinforcing the association between socioeconomic vulnerability and continued reliance on traditional practices.

The practice of traditional uvulectomy was strongly associated with demographic factors, including younger maternal age, lower educational level, higher parity, and adherence to tribal traditions. In this study, 92% of cases were linked to tribal customs, and all associations were statistically significant (p < 0.001). These findings are consistent with previous reports linking traditional medical practices to social structure and limited access to formal healthcare [20,23,31]. Multivariable analysis confirmed that parity and education remained independent predictors, reinforcing the role of social context rather than individual belief alone.

Traditional uvulectomy was commonly performed by non-medical practitioners using unsterilized instruments such as flat irons (25%), nail hooks (18%), or razor blades (14%). Comparable practices have been documented in Ethiopia and Uganda [3,26]. These unsafe techniques contribute to significant morbidity and reflect the influence of socioeconomic constraints and cultural norms. Notably, the persistence of the practice in urban settings indicates that cultural adherence outweighs geographical access to healthcare.

Ugandan studies report unsterilized instruments (razor blades, sticks) as major complication contributors [31]. Unsafe techniques have been associated with hemorrhage leading to anemia in newborns [32], and bleeding remains the most commonly reported complication across studies [16,17,33]. In our study, fever (35%), feeding difficulties (21%), and bleeding (13%) were the most frequently reported complications. Tetanus (1.2%) and infection (4.8%) were also observed, indicating that serious morbidity continues to occur despite the perception that the procedure is benign.

Hospitalization due to hemorrhage was required in 12% of cases in this study. This rate is considerably lower than those reported in Nigeria, where hospitalization rates of 85.7% in Sokoto have been documented [27]. Fever was reported in 34% of cases in the present study, while feeding difficulties affected 21% of children, findings that are comparable to those reported in Sokoto, Nigeria (28%) [27]. Infection rates were similarly low, at 2.9%, yet still clinically significant given the potential severity of post-procedural infections. Although septicemia remains relatively uncommon, it is a potentially life-threatening complication associated with traditional uvulectomy [23].

Traditional practitioners frequently employ local remedies such as acacia and rose geranium during the procedure. Evidence from Danfodiyo University Teaching Hospital in Sokoto, Nigeria, indicates that 33.3% of traditional healers administer medications to control post-procedural bleeding [27]. Despite these practices, awareness of complications remains limited. In the present study, 57% of mothers were unaware of potential complications, and 49% continued breastfeeding following the procedure, reflecting normalization of associated risks. This normalization underscores the urgent need for culturally sensitive health education and complication-prevention strategies delivered in local languages and community contexts.

### Recommendations

Based on the findings of this study, reducing the practice of traditional uvulectomy in Sudan requires culturally sensitive, multi-level interventions. Public health strategies should prioritize community-based education that directly addresses misconceptions about the function of the uvula and the risks associated with its removal, using locally appropriate languages and communication channels. Given the influential role of mothers and grandmothers, targeted engagement of these groups along with traditional leaders and healers should be central to behavior-change interventions. Strengthening the capacity of primary healthcare providers to offer culturally respectful counseling and safe alternatives for managing common childhood symptoms is essential to rebuild trust in formal health services. Integrating community dialogue, maternal education, and health worker training into existing maternal and child health programs may enhance acceptability and sustainability. In parallel, policy efforts should support regulation and monitoring of harmful traditional practices while avoiding punitive approaches that may drive them underground.

## Conclusion

This study demonstrates that traditional uvulectomy remains a deeply embedded cultural practice among Sudanese children and adolescents, driven by maternal beliefs, intergenerational influence, and tribal identity. Despite increasing educational attainment and access to healthcare, cultural norms frequently override biomedical knowledge. The continued use of unsafe techniques and the persistence of preventable complications highlight an urgent need for culturally grounded interventions. Sustainable reduction of this harmful practice requires community-engaged strategies that integrate health education, cultural dialogue, and strengthened healthcare services while respecting local social structures.

### Study strengths and limitations

This study benefits from a large, multi-regional community-based sample, enhancing its generalizability and relevance. The inclusion of detailed sociocultural and behavioral variables provides valuable insight into the drivers of traditional uvulectomy beyond clinical factors. However, the cross-sectional design limits causal inference, and reliance on self-reported data introduces the potential for recall and social desirability bias. Additionally, qualitative exploration of belief systems was beyond the scope of this study but warrants future investigation.

### Ethical Approval

This study was conducted in accordance with the Declaration of Helsinki. Ethical approval was obtained from the General Administration of Health Systems and Research at the Federal Ministry of Health, River Nile State, Sudan.

### Informed Consent

Written informed consent was obtained from all adult participants and from the parents or legal guardians of children and adolescents included in the study. All participants were informed of the study’s purpose, and confidentiality was strictly maintained through the use of anonymized data collection tools.
