# Supplementary material for: Prevalence, practices, and maternal perceptions of traditional uvulectomy among sudanese children and adolescents: A community-based cross-sectional study
Source: PLoS One. 2026 Jul 16;21(7):e0328341. doi: 10.1371/journal.pone.0328341 (PMC13374886; doi:10.1371/journal.pone.0328341)
Supplement: Attachment — Submitted filename: resonses of the reveiwer.docx [file pone.0328341.s002.docx]

Response to Reviewers,

We sincerely thank the editor and all reviewers for their careful evaluation of our manuscript and for their constructive and insightful comments. We have revised the manuscript extensively in response, particularly the Abstract, Introduction, and Methods, to improve clarity, rigor, and alignment with journal guidelines. Our detailed responses are provided below.

**1. Abstract**

Reviewer Comments

Background lacks a clear knowledge gap and adverse consequences

Study design is not clearly stated

Objectives are partially missing

Associated factors and analytical approach are unclear

Results are inadequate or inconsistent with objectives

Confidence intervals were not reported

Conclusion is weak and requires revision

Terminology and wording require improvement

Response

The Abstract has been completely revised to address these concerns. Specifically:

A clear knowledge gap is now articulated, highlighting the lack of national, multi-state data on traditional uvulectomy and maternal perceptions in Sudan.

The study design is explicitly stated as a community-based cross-sectional study.

The objectives now clearly include prevalence estimation, maternal perceptions, procedural practices, and associated risk factors.

The statistical methods used for identifying associated factors are summarized.

Key results are aligned with the objectives and presented clearly.

The conclusion has been strengthened to emphasize public health implications and the need for culturally sensitive interventions.

Language has been edited to ensure clarity, precision, and originality, avoiding phrasing that may appear AI-generated.

**2. Introduction**

Reviewer Comments

Introduction is inadequate and poorly structured

Magnitude, severity, and associated factors are missing

Global, regional, and national context is insufficient

Prevention and control efforts are not discussed

Objectives are not clearly stated at the end of the section

Grammatical and stylistic issues present

Response

The Introduction has been expanded and reorganized:

Added a detailed description of the magnitude and severity of traditional uvulectomy as a harmful traditional practice affecting child health.

Included global, regional, and Sudan-specific evidence on prevalence, complications, and cultural drivers.

Discussed socioeconomic, educational, and cultural factors associated with the persistence of the practice.

Briefly reviewed existing efforts to prevent harmful traditional practices, highlighting gaps in oral and paediatric health interventions.

Clearly stated the study objectives at the end of the Introduction.

Corrected grammatical errors and improved sentence structure for clarity and academic tone.

**3. Methods**

Reviewer Comments

Section organization is poor and inconsistent with journal guidelines

Study setting is inadequately described

Justification for convenience sampling is missing

Data collection tools and validation are unclear

Local language and translation process not specified

Data quality assurance procedures not described

Characteristics and training of data collectors not reported

Response

The Methods section has been thoroughly revised and reorganized:

Renamed to “Methods” and structured according to journal guidelines.

Expanded the study setting description, including participating states and community context.

Provided a clear justification for convenience sampling, considering security challenges, population displacement, and access limitations.

Described the questionnaire in detail, including source, domains, adaptation from previous studies, and pilot testing.

Specified the local languages used and explained the translation and back-translation process.

Added a detailed data quality assurance subsection, including training, supervision, and consistency checks.

Clarified the number, academic level, and training of medical student data collectors involved in the study.

**Statistical Analysis and Data Presentation**

Reviewer Comments

**Add Confidence Intervals: For all percentages reported, especially in the abstract, you must add the corresponding 95% Confidence Intervals (CI). (Reviewer #2)**

Response

Confidence intervals were added along with p values

Reviewer Comments

**Report Risk Factor Analysis: The abstract and the main results section are missing the outputs of the risk factor analysis. You must present the Odds Ratios (OR) or Adjusted Odds Ratios (AOR) from your regression models. (Reviewer #2).**

Response

Multiple logistic regression model with adjusted odds ratios was added to the results

Reviewer Comments

**Re-check All Statistics: Reviewer #2 explicitly requests that you "Check for statistics,".**

Response

We used Pearson's Chi-squared test or Fisher's exact test when appropriate to test the associations of categorical variables with our binary outcome. For continuous variables, we used Wilcoxon rank-sum test because our continuous variables were not normally distributed. Wilcoxon rank-sum test (also known as the Mann-Whitney U test) is specifically designed for comparing two independent groups when the data is not normally distributed.

Reviewer Comments

**Perform and Present Subgroup Analyses: The results are considered too general. You need to conduct and report subgroup analyses for key outcomes based on: Child's age groups (e.g., <5, 6-10, etc.). Child's sex. Maternal age (e.g., <35 and >35 years). Whether the mothers sought medical care. (Reviewer #2)**

Response

Table 2 was updated to include maternal age as well as child’s sex & age

**2. Clarity and Content of Results**

Reviewer Comments

**Expand the Results Section: The section is currently "too short." You need to provide a more detailed narrative of your findings, guided by the new analyses recommended above. (Reviewer #2).**

Response

A more detailed updated interpretation was included

Reviewer Comments

**Clarify Maternal Perception Discrepancy: There is a noted inconsistency in your findings. You must address and clarify why 30% of mothers believe uvulectomy is beneficial while 47% believe the uvula is not harmful. This may require presenting the data in a way that explains this apparent contradiction. (Reviewer #1)**

Response

After categorizing the mothers’ believe about uvulectomy, by the if they had a child with his/her uvula removed or not, it is observed that uvulectomy practice is significantly associated with believe

Reviewer Comments

**Clarify Basis for "Reasons for Uvulectomy": In Table 3, you must clarify whether the "Reasons for uvulectomy practice" are based on the general knowledge of all mothers or only from mothers whose children have actually undergone the procedure. (Reviewer #3)**

Response

Yes, you are right. Perceived reasons differ in mothers whose children have actually undergone the procedure. We updated table 3.

Reviewer Comments

**Provide Detail on Decision-Makers: Regarding Table 4, you should report if the decision to perform a uvulectomy was made by more than one person (e.g., both parents, mother and grandmother). This may require adding a new result if the data is available. (Reviewer #3)**

Response

Combinations of the influencers of uvulectomy practice were visualized and added to the results

Reviewer Comments

**Clarify Variable Categorization: You need to clarify how certain items, like "Acacia (Qarad)" and "Rose geraniums (a'atroon)," were categorized. State clearly if they were treated as medications or traditional remedies in your analysis. (Reviewer #3).**

Response

Yes, they should be treated as traditional remedies

Reviewer Comments

**Specify Context of Uvulectomy: The results should be more specific. Clarify if the practice was performed on any child suffering from a relevant ailment, or only after one or more occurrences of symptoms. (Reviewer #2)**

Response

This is good recommendation, but, unfortunately, this information is missing in the available data

**3. Table and Figure Modifications**

Reviewer Comments

**Make Tables Self-Explanatory: The tables are not clear on their own. Improve table titles and footnotes to ensure they can be understood without referring to the main text. (Reviewer #2)**

Response

Tables aesthetics were improved.

Reviewer Comments

**Condense and Refine Tables: The tables are excessively long ("about nine pages"). You must condense them by combining or removing variables. Specifically, the variable "Where are you from?" was suggested for removal. (Reviewer #3)**

Response

Suggested variables were removed

Reviewer Comments

**Add Illustratives (Figures/Graphs): The results section relies solely on text and tables. You are advised to "Use illustratives." Consider creating figures or graphs (e.g., bar charts for prevalence, forest plots for risk factors) to visually represent key findings. (Reviewer #2)**

Response

Two illustrative figures were added to the results

Reviewer Comments

**Reference Table 5 in the Text: You have a table (Table 5) that is not cited in the manuscript text. You must add a reference to this table within the results narrative. (Journal Requirements)**

Response

We referred to table 5 in the results section

Discussion, Recommendations, and Limitations

Reviewer Comments

There appears to be an inconsistency in the findings regarding maternal perceptions of uvulectomy. While 30% of respondents believe uvulectomy is a beneficial treatment, a larger proportion (47%) indicated that the presence of the uvula is not harmful to the child. Please address this discrepancy in the discussion.

Response

We thank the reviewer for this observation and have clarified this issue in the revised Discussion section

Reviewer Comments

The discussion requires more detailed explanation, justification, and comparison with existing studies.

Response

We have substantially expanded the Discussion to provide deeper interpretation and to compare our results with previous studies from Sudan, Ethiopia, and Nigeria . This comparative framing highlights how our findings align with regional data and contribute novel insights regarding perception-driven practice continuity.

Reviewer Comments

The reviewer requested that the authors better interpret their own findings.

Response

Following this suggestion, we provided a more detailed interpretation of our main odds ratios (OR 60.0, 6.38, and 0.24), describing both statistical significance and plausible explanatory mechanisms

Reviewer Comments

The recommendations are inflated.

Response

We have carefully revised and condensed the recommendations into a concise four-point prioritized list .

Reviewer Comments

The limitations section is incomplete.

Response

The Limitations section has been revised to include an explicit account of methodological constraints .

We now acknowledge that the use of convenience sampling may introduce selection bias, potentially limiting representativeness of the broader population. Additionally, as responses were self-reported, the possibility of recall and social desirability bias has been recognized, especially regarding mothers’ disclosure of traditional practices. These revisions improve the transparency and realism of our methodological interpretation.
