# Supplementary material for: Prevalence, practices, and maternal perceptions of traditional uvulectomy among sudanese children and adolescents: A community-based cross-sectional study
Source: PLoS One. 2026 Jul 16;21(7):e0328341. doi: 10.1371/journal.pone.0328341 (PMC13374886; doi:10.1371/journal.pone.0328341)
Supplement: Peer review history

# Peer review: Decision Letter 0

**DOI:** 10.1371/journal.pone.0328341.r001

**Authors:** Kahsu Gebrekidan

19 Nov 2025

PONE-D-25-35262

Prevalence and Practice of Traditional Uvulectomy Among Sudanese Children and Adolescents, Along with Maternal Perception and Associated Risk Factors: A Community-Based Cross-Sectional Study

PLOS ONE

Dear Dr. Jaber,

Thank you for submitting your manuscript to PLOS ONE. After careful consideration, we feel that it has merit but does not fully meet PLOS ONE’s publication criteria as it currently stands. Therefore, we invite you to submit a revised version of the manuscript that addresses the points raised during the review process.

Please submit your revised manuscript by Jan 03 2026 11:59PM. If you will need more time than this to complete your revisions, please reply to this message or contact the journal office at plosone@plos.org. When you're ready to submit your revision, log on to https://www.editorialmanager.com/pone/ and select the 'Submissions Needing Revision' folder to locate your manuscript file.

Please include the following items when submitting your revised manuscript:

- A rebuttal letter that responds to each point raised by the academic editor and reviewer(s). You should upload this letter as a separate file labeled 'Response to Reviewers'.
- A marked-up copy of your manuscript that highlights changes made to the original version. You should upload this as a separate file labeled 'Revised Manuscript with Track Changes'.
- An unmarked version of your revised paper without tracked changes. You should upload this as a separate file labeled 'Manuscript'.

If you would like to make changes to your financial disclosure, please include your updated statement in your cover letter. Guidelines for resubmitting your figure files are available below the reviewer comments at the end of this letter.

If applicable, we recommend that you deposit your laboratory protocols in protocols.io to enhance the reproducibility of your results. Protocols.io assigns your protocol its own identifier (DOI) so that it can be cited independently in the future. For instructions see: https://journals.plos.org/plosone/s/submission-guidelines#loc-laboratory-protocols. Additionally, PLOS ONE offers an option for publishing peer-reviewed Lab Protocol articles, which describe protocols hosted on protocols.io. Read more information on sharing protocols at https://plos.org/protocols?utm_medium=editorial-email&utm_source=authorletters&utm_campaign=protocols.

We look forward to receiving your revised manuscript.

Kind regards,

Kahsu Gebrekidan, Ph.D.

Academic Editor

PLOS ONE

Journal Requirements:

When submitting your revision, we need you to address these additional requirements.

1. Please ensure that your manuscript meets PLOS ONE's style requirements, including those for file naming. The PLOS ONE style templates can be found at

https://journals.plos.org/plosone/s/file?id=wjVg/PLOSOne_formatting_sample_main_body.pdf and

https://journals.plos.org/plosone/s/file?id=ba62/PLOSOne_formatting_sample_title_authors_affiliations.pdf.

2. We suggest you thoroughly copyedit your manuscript for language usage, spelling, and grammar. If you do not know anyone who can help you do this, you may wish to consider employing a professional scientific editing service.

The American Journal Experts (AJE) (https://www.aje.com/) is one such service that has extensive experience helping authors meet PLOS guidelines and can provide language editing, translation, manuscript formatting, and figure formatting to ensure your manuscript meets our submission guidelines. Please note that having the manuscript copyedited by AJE or any other editing services does not guarantee selection for peer review or acceptance for publication.

Upon resubmission, please provide the following:

The name of the colleague or the details of the professional service that edited your manuscript

A copy of your manuscript showing your changes by either highlighting them or using track changes (uploaded as a *supporting information* file)

A clean copy of the edited manuscript (uploaded as the new *manuscript* file)”

3. We note that your Data Availability Statement is currently as follows: [All relevant data is

within the paper.]

Please confirm at this time whether or not your submission contains all raw data required to replicate the results of your study. Authors must share the “minimal data set” for their submission. PLOS defines the minimal data set to consist of the data required to replicate all study findings reported in the article, as well as related metadata and methods (https://journals.plos.org/plosone/s/data-availability#loc-minimal-data-set-definition).

For example, authors should submit the following data:

- The values behind the means, standard deviations and other measures reported;

- The values used to build graphs;

- The points extracted from images for analysis.

Authors do not need to submit their entire data set if only a portion of the data was used in the reported study.

If your submission does not contain these data, please either upload them as Supporting Information files or deposit them to a stable, public repository and provide us with the relevant URLs, DOIs, or accession numbers. For a list of recommended repositories, please see https://journals.plos.org/plosone/s/recommended-repositories.

If there are ethical or legal restrictions on sharing a de-identified data set, please explain them in detail (e.g., data contain potentially sensitive information, data are owned by a third-party organization, etc.) and who has imposed them (e.g., an ethics committee). Please also provide contact information for a data access committee, ethics committee, or other institutional body to which data requests may be sent. If data are owned by a third party, please indicate how others may request data access.

4. We note you have included a table to which you do not refer in the text of your manuscript. Please ensure that you refer to Table 5 in your text; if accepted, production will need this reference to link the reader to the Table

5. If the reviewer comments include a recommendation to cite specific previously published works, please review and evaluate these publications to determine whether they are relevant and should be cited. There is no requirement to cite these works unless the editor has indicated otherwise.

[Note: HTML markup is below. Please do not edit.]

Reviewers' comments:

Reviewer's Responses to Questions

**Comments to the Author**

1. Is the manuscript technically sound, and do the data support the conclusions?

The manuscript must describe a technically sound piece of scientific research with data that supports the conclusions. Experiments must have been conducted rigorously, with appropriate controls, replication, and sample sizes. The conclusions must be drawn appropriately based on the data presented.

Reviewer #1: Yes

Reviewer #2: Partly

Reviewer #3: Yes

**********

2. Has the statistical analysis been performed appropriately and rigorously?

Reviewer #1: Yes

Reviewer #2: No

Reviewer #3: Yes

**********

3. Have the authors made all data underlying the findings in their manuscript fully available?

The PLOS Data policy requires authors to make all data underlying the findings described in their manuscript fully available without restriction, with rare exception (please refer to the Data Availability Statement in the manuscript PDF file). The data should be provided as part of the manuscript or its supporting information, or deposited to a public repository. For example, in addition to summary statistics, the data points behind means, medians and variance measures should be available. If there are restrictions on publicly sharing data—e.g. participant privacy or use of data from a third party—those must be specified.

Reviewer #1: Yes

Reviewer #2: Yes

Reviewer #3: Yes

**********

4. Is the manuscript presented in an intelligible fashion and written in standard English?

PLOS ONE does not copyedit accepted manuscripts, so the language in submitted articles must be clear, correct, and unambiguous. Any typographical or grammatical errors should be corrected at revision, so please note any specific errors here.

Reviewer #1: Yes

Reviewer #2: Yes

Reviewer #3: Yes

**********

5. Review Comments to the Author

Please use the space provided to explain your answers to the questions above. You may also include additional comments for the author, including concerns about dual publication, research ethics, or publication ethics. (Please upload your review as an attachment if it exceeds 20,000 characters)

Reviewer #1: To the Editor:

Thank you for the opportunity to review this manuscript.

To the Author:

Overall, the work is well-executed and demonstrates a commendable dedication to community-level research. I do have some suggestions for improvement:

1. Please revise the manuscript to ensure all text reflects your own writing style and avoid the use of AI-generated language. Paraphrase any sections that may have been produced by AI.

2. Please format the author list with numerical superscripts to clearly indicate author affiliations or order.

3. There appears to be an inconsistency in the findings regarding maternal perceptions of uvulectomy. While 30% of respondents believe uvulectomy is a beneficial treatment, a larger proportion (47%) indicated that the presence of the uvula is not harmful to the child. Please address this discrepancy in the discussion. If a significant portion of participants do not believe the uvula poses a threat, explain the basis for the 30% who consider its removal to be a good treatment, particularly given the study's focus on maternal perceptions.

Reviewer #2: Review Report

Title :Prevalence and Practice of Traditional Uvulectomy Among Sudanese Children and Adolescents, Along with Maternal Perception and Associated Risk Factors: A Community-Based Cross-Sectional Study

Review Comments

A.General Comments

-Use appropriate guideline of the journal

-Add line numbers

-Inappropriate sentencing E.g. Introduction

-Subsections entail inadequate contents

-Grammatical errors

B.Specific Comments

i. Scope

-Is that practice or perception ? Is that single practice or repeatition or both? How do you analyze the practice with perception? oe indeendntly since both of them are maternal perceptions and practice?

ii. Title

-Rewrite it

-Add years

iii. Abstract

-The background fails to contain strong knowldge gap and advae consequence

-Method should be methods

-Use CI while stating the percentage

-Associated factors and the appropriate analysis is missed

-What unsterilized tools? What did they actually use?

-Not appropriate analysis is presented

-Partial objective is missed

-Study design is missed

-Results are indequate, inconsitent with the objective E.g. perception, not presented OR or AOR

-The conclusion is weak, rewrite it again,

iv. Introduction

-Inadequate

-Icomplete E.g. years missed

-Magnitude, severity, factis asociated, eforts made gobaly, reginaly and natinaly to prevent and control HTPs including traditiinal veloctomy is missed.

-Fine objectives shoud be presented at the end of the introduction section

v.Methodology

-Use methods and materials instead or maintain the guideline if the journal

-Poorly organized E.g. Sub sections are not wel orgnized

-Study setting :E.g. The ten stats, health services avaiable were missed

-Why you used convinience sampling in comunity based studies

-What is that local language? [translation]

-The tool, its types, source, contents, validation, the data quaity asurance means were missed? E.g. The quaity of numrators and whethe there is supervision is missed.

-What was done for those with UT?

vi.Results

-Is short

-Maintain comsitency

-Use ilusteratives

-Tables are not self explanatory

-Check for statistics

-Sub group analysis for age a <5, 6-10, 1 and more years can be dne, sex can alo better explain it. In additiin, maternal age can be classified as <35 and >35 years, Whether they conault for medical care or not. Again, for whom did they done TU? Is that whonwvr ufrs from it? Or among those whosufer it once? or more than once?

-Consult on how to write the result section [Refer to articles in the same journal]

Vii. Discusion and other consequent sections

-More detail exlpanatin, justificatin and refetrences should be used

-Consult your own findings for better explanations

-The cnclusion is not inline with the the scope

-Rec9mdatins are infated

Reviewer #3: Dear authors

It is a nice topic , unfortunately the habit Traditional uvulectomy still present in some countries beside my comments on this article , I think it is better to conduct other community based interventional study to formulate some strategies for stopping this habit

Title

The present title is “Prevalence and Practice of Traditional Uvulectomy Among Sudanese Children and Adolescents, Along with Maternal Perception and Associated Risk Factors: A Community-Based Cross-Sectional Study” The title is too long It is better to be shorten to “Prevalence and Practice of Traditional Uvulectomy Among Sudanese Children and Adolescents with Maternal Perception and Associated Risk Factors”

Methods

1. The data collected by trained medical students , How many students were participated and at which college’s level

2. The questionnaire used in the study prepared by the researchers or depended on the previous studies , and What are the local languages used?

3. Family income depended on which criteria?

4.

5.

Results

1. The tables are too long about nine pages

2. There are many variables in the tables , I think some can be combined , and others removed like the question “Where are you from?”

3. Clarify about “Reasons for uvulectomy practice” in the table 3 , these are depended on the mother’s knowledge or those mothers whom their children underwent uvulectomy

4. In table 4 , did you find the decision of doing uvulectomy done by more than one person could be both parents or the mother and grandmother and so on?

5. Do you regard the ( Acacia ( Qarad) ,- Rose geraniums ( a'atroon)) as medications ?

6.

Discussion

1. There is a is a poor / minimal comparison with previous (local or international studies) , References

Follow the journal guideline regarding references writing

1. Journal titles should be unified either in abbreviated form or in full title form

2. Only the first six authors surname and initials should be written and the suffix et al will be added for the seventh and above.

3. It is not necessary to add PMID , PMCID , Epub of the articles , only add the doi.

**********

6. PLOS authors have the option to publish the peer review history of their article (what does this mean?). If published, this will include your full peer review and any attached files.

If you choose “no”, your identity will remain anonymous but your review may still be made public.

**Do you want your identity to be public for this peer review?**  For information about this choice, including consent withdrawal, please see our Privacy Policy.

Reviewer #1: No

Reviewer #2: No

Reviewer #3: **Yes:** Masood Abdulkareem Abdulrahman

**********

[NOTE: If reviewer comments were submitted as an attachment file, they will be attached to this email and accessible via the submission site. Please log into your account, locate the manuscript record, and check for the action link "View Attachments". If this link does not appear, there are no attachment files.]

To ensure your figures meet our technical requirements, please review our figure guidelines: https://journals.plos.org/plosone/s/figures

You may also use PLOS’s free figure tool, NAAS, to help you prepare publication quality figures: https://journals.plos.org/plosone/s/figures#loc-tools-for-figure-preparation.

NAAS will assess whether your figures meet our technical requirements by comparing each figure against our figure specifications.

# Author comment: Author response to Decision Letter 1

**DOI:** 10.1371/journal.pone.0328341.r002

8 Mar 2026

Response to Reviewers,

We sincerely thank the editor and all reviewers for their careful evaluation of our manuscript and for their constructive and insightful comments. We have revised the manuscript extensively in response, particularly the Abstract, Introduction, and Methods, to improve clarity, rigor, and alignment with journal guidelines. Our detailed responses are provided below.

1. Abstract

Reviewer Comments

Background lacks a clear knowledge gap and adverse consequences

Study design is not clearly stated

Objectives are partially missing

Associated factors and analytical approach are unclear

Results are inadequate or inconsistent with objectives

Confidence intervals were not reported

Conclusion is weak and requires revision

Terminology and wording require improvement

Response

The Abstract has been completely revised to address these concerns. Specifically:

A clear knowledge gap is now articulated, highlighting the lack of national, multi-state data on traditional uvulectomy and maternal perceptions in Sudan.

The study design is explicitly stated as a community-based cross-sectional study.

The objectives now clearly include prevalence estimation, maternal perceptions, procedural practices, and associated risk factors.

The statistical methods used for identifying associated factors are summarized.

Key results are aligned with the objectives and presented clearly.

The conclusion has been strengthened to emphasize public health implications and the need for culturally sensitive interventions.

Language has been edited to ensure clarity, precision, and originality, avoiding phrasing that may appear AI-generated.

2. Introduction

Reviewer Comments

Introduction is inadequate and poorly structured

Magnitude, severity, and associated factors are missing

Global, regional, and national context is insufficient

Prevention and control efforts are not discussed

Objectives are not clearly stated at the end of the section

Grammatical and stylistic issues present

Response

The Introduction has been expanded and reorganized:

Added a detailed description of the magnitude and severity of traditional uvulectomy as a harmful traditional practice affecting child health.

Included global, regional, and Sudan-specific evidence on prevalence, complications, and cultural drivers.

Discussed socioeconomic, educational, and cultural factors associated with the persistence of the practice.

Briefly reviewed existing efforts to prevent harmful traditional practices, highlighting gaps in oral and paediatric health interventions.

Clearly stated the study objectives at the end of the Introduction.

Corrected grammatical errors and improved sentence structure for clarity and academic tone.

3. Methods

Reviewer Comments

Section organization is poor and inconsistent with journal guidelines

Study setting is inadequately described

Justification for convenience sampling is missing

Data collection tools and validation are unclear

Local language and translation process not specified

Data quality assurance procedures not described

Characteristics and training of data collectors not reported

Response

The Methods section has been thoroughly revised and reorganized:

Renamed to “Methods” and structured according to journal guidelines.

Expanded the study setting description, including participating states and community context.

Provided a clear justification for convenience sampling, considering security challenges, population displacement, and access limitations.

Described the questionnaire in detail, including source, domains, adaptation from previous studies, and pilot testing.

Specified the local languages used and explained the translation and back-translation process.

Added a detailed data quality assurance subsection, including training, supervision, and consistency checks.

Clarified the number, academic level, and training of medical student data collectors involved in the study.

Statistical Analysis and Data Presentation

Reviewer Comments

Add Confidence Intervals: For all percentages reported, especially in the abstract, you must add the corresponding 95% Confidence Intervals (CI). (Reviewer #2)

Response

Confidence intervals were added along with p values

Reviewer Comments

Report Risk Factor Analysis: The abstract and the main results section are missing the outputs of the risk factor analysis. You must present the Odds Ratios (OR) or Adjusted Odds Ratios (AOR) from your regression models. (Reviewer #2).

Response

Multiple logistic regression model with adjusted odds ratios was added to the results

Reviewer Comments

Re-check All Statistics: Reviewer #2 explicitly requests that you "Check for statistics,".

Response

We used Pearson's Chi-squared test or Fisher's exact test when appropriate to test the associations of categorical variables with our binary outcome. For continuous variables, we used Wilcoxon rank-sum test because our continuous variables were not normally distributed. Wilcoxon rank-sum test (also known as the Mann-Whitney U test) is specifically designed for comparing two independent groups when the data is not normally distributed.

Reviewer Comments

Perform and Present Subgroup Analyses: The results are considered too general. You need to conduct and report subgroup analyses for key outcomes based on: Child's age groups (e.g., <5, 6-10, etc.). Child's sex. Maternal age (e.g., <35 and >35 years). Whether the mothers sought medical care. (Reviewer #2)

Response

Table 2 was updated to include maternal age as well as child’s sex & age

2. Clarity and Content of Results

Reviewer Comments

Expand the Results Section: The section is currently "too short." You need to provide a more detailed narrative of your findings, guided by the new analyses recommended above. (Reviewer #2).

Response

A more detailed updated interpretation was included

Reviewer Comments

Clarify Maternal Perception Discrepancy: There is a noted inconsistency in your findings. You must address and clarify why 30% of mothers believe uvulectomy is beneficial while 47% believe the uvula is not harmful. This may require presenting the data in a way that explains this apparent contradiction. (Reviewer #1)

Response

After categorizing the mothers’ believe about uvulectomy, by the if they had a child with his/her uvula removed or not, it is observed that uvulectomy practice is significantly associated with believe

Reviewer Comments

Clarify Basis for "Reasons for Uvulectomy": In Table 3, you must clarify whether the "Reasons for uvulectomy practice" are based on the general knowledge of all mothers or only from mothers whose children have actually undergone the procedure. (Reviewer #3)

Response

Yes, you are right. Perceived reasons differ in mothers whose children have actually undergone the procedure. We updated table 3.

Reviewer Comments

Provide Detail on Decision-Makers: Regarding Table 4, you should report if the decision to perform a uvulectomy was made by more than one person (e.g., both parents, mother and grandmother). This may require adding a new result if the data is available. (Reviewer #3)

Response

Combinations of the influencers of uvulectomy practice were visualized and added to the results

Reviewer Comments

Clarify Variable Categorization: You need to clarify how certain items, like "Acacia (Qarad)" and "Rose geraniums (a'atroon)," were categorized. State clearly if they were treated as medications or traditional remedies in your analysis. (Reviewer #3).

Response

Yes, they should be treated as traditional remedies

Reviewer Comments

Specify Context of Uvulectomy: The results should be more specific. Clarify if the practice was performed on any child suffering from a relevant ailment, or only after one or more occurrences of symptoms. (Reviewer #2)

Response

This is good recommendation, but, unfortunately, this information is missing in the available data

3. Table and Figure Modifications

Reviewer Comments

Make Tables Self-Explanatory: The tables are not clear on their own. Improve table titles and footnotes to ensure they can be understood without referring to the main text. (Reviewer #2)

Response

Tables aesthetics were improved.

Reviewer Comments

Condense and Refine Tables: The tables are excessively long ("about nine pages"). You must condense them by combining or removing variables. Specifically, the variable "Where are you from?" was suggested for removal. (Reviewer #3)

Response

Suggested variables were removed

Reviewer Comments

Add Illustratives (Figures/Graphs): The results section relies solely on text and tables. You are advised to "Use illustratives." Consider creating figures or graphs (e.g., bar charts for prevalence, forest plots for risk factors) to visually represent key findings. (Reviewer #2)

Response

Two illustrative figures were added to the results

Reviewer Comments

Reference Table 5 in the Text: You have a table (Table 5) that is not cited in the manuscript text. You must add a reference to this table within the results narrative. (Journal Requirements)

Response

We referred to table 5 in the results section

Discussion, Recommendations, and Limitations

Reviewer Comments

There appears to be an inconsistency in the findings regarding maternal perceptions of uvulectomy. While 30% of respondents believe uvulectomy is a beneficial treatment, a larger proportion (47%) indicated that the presence of the uvula is not harmful to the child. Please address this discrepancy in the discussion.

Response

We thank the reviewer for this observation and have clarified this issue in the revised Discussion section

Reviewer Comments

The discussion requires more detailed explanation, justification, and comparison with existing studies.

Response

We have substantially expanded the Discussion to provide deeper interpretation and to compare our results with previous studies from Sudan, Ethiopia, and Nigeria . This comparative framing highlights how our findings align with regional data and contribute novel insights regarding perception-driven practice continuity.

Reviewer Comments

The reviewer requested that the authors better interpret their own findings.

Response

Following this suggestion, we provided a more detailed interpretation of our main odds ratios (OR 60.0, 6.38, and 0.24), describing both statistical significance and plausible explanatory mechanisms

Reviewer Comments

The recommendations are inflated.

Response

We have carefully revised and condensed the recommendations into a concise four-point prioritized list .

Reviewer Comments

The limitations section is incomplete.

Response

The Limitations section has been revised to include an explicit account of methodological constraints .

We now acknowledge that the use of convenience sampling may introduce selection bias, potentially limiting representativeness of the broader population. Additionally, as responses were self-reported, the possibility of recall and social desirability bias has been recognized, especially regarding mothers’ disclosure of traditional practices. These revisions improve the transparency and realism of our methodological interpretation.

Attachment Submitted filename: resonses of the reveiwer.docx

# Peer review: Decision Letter 1

**DOI:** 10.1371/journal.pone.0328341.r003

**Authors:** Kahsu Gebrekidan, Kahsu Gebrekidan

2 Jun 2026

Prevalence, Practices, and Maternal Perceptions of Traditional Uvulectomy Among Sudanese Children and Adolescents: A Community-Based Cross-Sectional Study

PONE-D-25-35262R1

Dear Dr. Jaber,

We’re pleased to inform you that your manuscript has been judged scientifically suitable for publication and will be formally accepted for publication once it meets all outstanding technical requirements.

Within one week, you’ll receive an e-mail detailing the required amendments. When these have been addressed, you’ll receive a formal acceptance letter and your manuscript will be scheduled for publication.

An invoice will be generated when your article is formally accepted. Please note, if your institution has a publishing partnership with PLOS and your article meets the relevant criteria, all or part of your publication costs will be covered. Please make sure your user information is up-to-date by logging into Editorial Manager at Editorial Manager® and clicking the ‘Update My Information' link at the top of the page. For questions related to billing, please contact billing support.

If your institution or institutions have a press office, please notify them about your upcoming paper to help maximize its impact. If they’ll be preparing press materials, please inform our press team as soon as possible -- no later than 48 hours after receiving the formal acceptance. Your manuscript will remain under strict press embargo until 2 pm Eastern Time on the date of publication. For more information, please contact onepress@plos.org.

Kind regards,

Kahsu Gebrekidan, Ph.D.

Academic Editor

PLOS One

Additional Editor Comments (optional):

Reviewers' comments:

Reviewer's Responses to Questions

**Comments to the Author**

1. If the authors have adequately addressed your comments raised in a previous round of review and you feel that this manuscript is now acceptable for publication, you may indicate that here to bypass the “Comments to the Author” section, enter your conflict of interest statement in the “Confidential to Editor” section, and submit your "Accept" recommendation.

Reviewer #1: All comments have been addressed

**********

2. Is the manuscript technically sound, and do the data support the conclusions?

The manuscript must describe a technically sound piece of scientific research with data that supports the conclusions. Experiments must have been conducted rigorously, with appropriate controls, replication, and sample sizes. The conclusions must be drawn appropriately based on the data presented.

Reviewer #1: Yes

**********

3. Has the statistical analysis been performed appropriately and rigorously?

Reviewer #1: Yes

**********

4. Have the authors made all data underlying the findings in their manuscript fully available?

The PLOS Data policy requires authors to make all data underlying the findings described in their manuscript fully available without restriction, with rare exception (please refer to the Data Availability Statement in the manuscript PDF file). The data should be provided as part of the manuscript or its supporting information, or deposited to a public repository. For example, in addition to summary statistics, the data points behind means, medians and variance measures should be available. If there are restrictions on publicly sharing data—e.g. participant privacy or use of data from a third party—those must be specified.

Reviewer #1: Yes

**********

5. Is the manuscript presented in an intelligible fashion and written in standard English?

PLOS ONE does not copyedit accepted manuscripts, so the language in submitted articles must be clear, correct, and unambiguous. Any typographical or grammatical errors should be corrected at revision, so please note any specific errors here.

Reviewer #1: Yes

**********

6. Review Comments to the Author

Please use the space provided to explain your answers to the questions above. You may also include additional comments for the author, including concerns about dual publication, research ethics, or publication ethics. (Please upload your review as an attachment if it exceeds 20,000 characters)

Reviewer #1: (No Response)

**********

7. PLOS authors have the option to publish the peer review history of their article (what does this mean?). If published, this will include your full peer review and any attached files.

If you choose “no”, your identity will remain anonymous but your review may still be made public.

**Do you want your identity to be public for this peer review?**  For information about this choice, including consent withdrawal, please see our Privacy Policy.

Reviewer #1: No

**********

# Editor report: Acceptance letter

**DOI:** 10.1371/journal.pone.0328341.r004

**Authors:** Kahsu Gebrekidan, Kahsu Gebrekidan

PONE-D-25-35262R1

PLOS One

Dear Dr. Jaber Amin,

I'm pleased to inform you that your manuscript has been deemed suitable for publication in PLOS One. Congratulations! Your manuscript is now being handed over to our production team.

At this stage, our production department will prepare your paper for publication. This includes ensuring the following:

* All references, tables, and figures are properly cited

* All relevant supporting information is included in the manuscript submission,

* There are no issues that prevent the paper from being properly typeset

You will receive further instructions from the production team, including instructions on how to review your proof when it is ready. Please keep in mind that we are working through a large volume of accepted articles, so please give us a few days to review your paper and let you know the next and final steps.

Lastly, if your institution or institutions have a press office, please let them know about your upcoming paper now to help maximize its impact. If they'll be preparing press materials, please inform our press team within the next 48 hours. Your manuscript will remain under strict press embargo until 2 pm Eastern Time on the date of publication. For more information, please contact onepress@plos.org.

You will receive an invoice from PLOS for your publication fee after your manuscript has reached the completed accept phase. If you receive an email requesting payment before acceptance or for any other service, this may be a phishing scheme. Learn how to identify phishing emails and protect your accounts at https://explore.plos.org/phishing.

If we can help with anything else, please email us at customercare@plos.org.

Thank you for submitting your work to PLOS ONE and supporting open access.

Kind regards,

PLOS ONE Editorial Office Staff

on behalf of

Dr. Kahsu Gebrekidan

Academic Editor

PLOS One
